# Reduced T2* Values in Soleus Muscle of Patients with Type 2 Diabetes Mellitus

**DOI:** 10.1371/journal.pone.0049337

**Published:** 2012-11-26

**Authors:** Chun S. Zuo, Young-Hoon Sung, Donald C. Simonson, Erin Habecker, Jian Wang, Charlotte Haws, Rosemond A. Villafuerte, Michael E. Henry, Robert L. Dobbins, Rebecca J. Hodge, Derek J. R. Nunez, Perry F. Renshaw

**Affiliations:** 1 Brain Imaging Center, McLean Hospital, Harvard Medical School, Belmont, Massachusetts, United States of America; 2 Brigham and Women's Hospital, Harvard Medical School, Boston, Massachusetts, United States of America; 3 Department of Radiology, Southwest Hospital, Third Military Medical University, Chongqing, China; 4 GlaxoSmithKline, Research and Development, Research Triangle Park, North Carolina, United States of America; Mayo Clinic, United States of America

## Abstract

Tissue water transverse relaxation times (T2) are highly sensitive to fluid and lipid accumulations in skeletal muscles whereas the related T2* is sensitive to changes in tissue oxygenation in addition to factors affecting T2. Diabetes mellitus (DM) affects muscles of lower extremities progressively by impairing blood flow at the macrovascular and microvascular levels. This study is to investigate whether T2 and T2* are sensitive enough to detect abnormalities in skeletal muscles of diabetic patients in the resting state. T2 and T2* values in calf muscle of 18 patients with type 2 DM (T2DM), 22 young healthy controls (YHC), and 7 age-matched older healthy controls (OHC) were measured at 3T using multi-TE spin echo and gradient echo sequences. Regional lipid levels of the soleus muscle were also measured using the Dixon method in a subset of the subjects. Correlations between T2, T2*, lipid levels, glycated hemoglobin (HbA1c) and presence of diabetes were evaluated. We found that T2 values were significantly higher in calf muscles of T2DM subjects, as were T2* values in anterior tibialis, and gastrocnemius muscles of T2DM participants. However, soleus T2* values of the T2DM subjects were significantly lower than those of the older, age-matched HC cohort (22.9±0.5 vs 26.7±0.4 ms, p<0.01). The soleus T2* values in the T2DM cohort were inversely correlated with the presence of diabetes (t = −3.46, p<0.001) and with an increase in HbA1c, but not with body mass index or regional lipid levels. Although multiple factors may contribute to changes in T2* values, the lowered T2* value observed in the T2DM soleus muscle is most consistent with a combination of high oxygen consumption and poor regional perfusion. This finding is consistent with results of previous perfusion studies and suggests that the soleus in individuals with T2DM is likely under tissue oxygenation stress.

## Introduction

Tissue oxygenation is vitally important for the metabolism and function of skeletal muscles [1]. Type 2 diabetes mellitus (T2DM) affects muscles, typically in the lower extremities, by impairing blood flow at the macrovascular and microvascular levels [2]. These changes are often relatively gradual and subtle, and the onset of clinical symptoms and deficits may be difficult to recognize until very late in the disease process, resulting in neuropathy and diabetic muscle infarction. Diagnosis of these early changes may require not only imaging techniques such as radiography and MR angiography to delineate the peripheral macrovasculature and to localize occlusions, but also imaging methods that provide additional information regarding tissue oxygenation, which reflects the balance between oxygen supply and demand in the deep muscles of the limbs.

Magnetic resonance imaging (MRI) methods have been developed to assess cerebral blood oxygenation non-invasively. Numerous studies have shown that T2* MRI is highly sensitive to the level of deoxyhemoglobin in blood [3], [4], [5] and, hence, it has been utilized to detect the blood oxygenation level dependent (BOLD) effects in the brain and other tissues [6], [7], [8]. BOLD signal intensity changes have been used as surrogate measures of oxygenation of blood and tissue to study a variety of physiological and pathological conditions associated with changes in oxygenation [5], [9], [10], [11]. Depending on the paradigm, changes in T2* values have also been used as surrogate measures of changes in oxygenation of blood and tissue [11], [12], [13]. It is generally accepted that BOLD MRI is sensitive to the concentration of paramagnetic deoxyhemoglobin, and thus indirectly to the relative oxyhemoglobin content at the microvascular level. The BOLD signal is also influenced by changes in perfusion, oxygen extraction rate, and blood volume or fluid level in the region of interest [14].

BOLD MRI of the skeletal muscle has recently been undertaken in healthy volunteers and patients [7], [15], [16], [17]. To date, various dynamic paradigms have been used to produce measurable BOLD signal alterations based on the assumption that the muscle BOLD response also originates mainly from changes in tissue oxygenation [7], [16]. While these challenge paradigms are very useful in model studies of young and middle aged subjects, their application is limited in the clinical setting due, in part, to variation in individual responses as well as weakness in older patients or in those with poor health conditions. Since diabetic patients are commonly found to have poor blood perfusion in their lower extremities, we hypothesized that T2* MRI would be sensitive enough to detect changes due to underlying physiological conditions in the lower extremities even in the resting state. In this preliminary study, we tested the hypothesis by measuring T2* values in the resting state instead of measuring BOLD signal changes between resting and ischemia/reperfusion states. We are interested in the resting state because most patients can tolerate resting state MRI scanning with minimal difficulties and because the methods are suitable for use in clinical practice. Our cross-sectional study in healthy controls and patients with type 2 diabetes mellitus (T2DM) focused on the soleus muscle because of its rich microvasculature and mitochondria, its essential role in lower limb function, and its high susceptibility to the pathophysiological effects of T2DM [18], [19].

## Materials and Methods

### Subjects

The study was conducted under protocols approved by The McLean Hospital Institutional Review Board (IRB). All subjects gave written informed consent before participating and had no contraindications to MRI. Three groups of subjects, i) eighteen subjects with T2DM (seventeen males and one female, mean±SD age: 54±12 yrs, body weight: 87.4±7.3 kg (BMI 23.8–34.3, duration of T2DM: 8.9±7.6 yrs), ii) twenty two young healthy male subjects (n = 22, age: 29±10 yrs, bodyweight 74±8.0 kg (BMI 20.3–28.9)), and iii) seven age-matched healthy subjects (n = 7, age 56±3 yrs, body weight 74.8±10.7 kg (BMI 22.2–27.9)), were recruited for comparison (Table 1). None of the T2DM subjects had medical or family histories of occlusive vascular disease in the legs, cardiovascular disease, clinical evidence of edema, diabetic neuropathy, renal dysfunction, muscle infarction or injury to the lower extremities. They were able to walk without difficulty during their screen visits and scan visits. Blood samples were also obtained for evaluation of long-term glycemic control using HbA1c. In addition, circumference of the calf muscles was also measured (see Table 1). To standardize the resting state, subjects were required to sit still for one hour with their feet resting gently on the floor prior to the MR scan.

**Table 1:** Summary of the subject information including age, BMI, HbA1c, and circumference of the calf.

	n	age (yrs)	BMI	HbA1c	Calf circumference
Young Healthy Control	22	29±10	23.9±2.4	5.36±0.33	38.3±2.7
Old Healthy Control	7	56±3	24.7±1.6	5.59±0.17	38.4±2.8
T2DM	18	54±12	30.5±3.3	7.7±1.2	40.2±2.7

### MRI

The MR images were acquired on a 3T MR scanner (Trio, Siemens AG, Germany) using a volume coil (inner diameter 27.3 cm) for transmission and reception. The subjects were placed in a supine position with their lower legs centered within the coil at the location of greatest circumference and imaged. In one T2DM subject, images were acquired from only one leg using a knee coil his legs were too large to be accommodated simultaneously in the volume coil. As mentioned in the introduction that T2* signals may also be influenced by factors other than level of blood oxygenation, T2 measurement was added as a control to help with the interpretation of possible T2* changes. T2, the transverse relaxation time, and T2* are related via the relationship 1/T2* = 1/T2+1/T2′, where T2′ is the component due to microscopic field gradients. T2 is sensitive to levels of fluid and lipid in the calf muscles. Proton T2 and T2* values were measured at identical locations with a multi-TE spin echo scheme (n = 12, TE1 = 15 ms, TE step 15 ms, total data acquisition time 5 minutes and 6 seconds) and a multi-TE gradient echo sequence (n = 12, TE1 = 9 ms, TE step = 3 ms, total data acquisition time 1 minute and 19 seconds), respectively.

After reviewing the linearity of log(s) vs TE plot, T2 and T2* maps were constructed using least square fits from multi-TE spin echo images and multi-TE gradient echo images, respectively, based on the relationship ln(s/so) = −TE/T2. The T2* fits were also made between the echoes at in-phase TEs (even multiples of 1.23 ms) and opposed-phase TEs (odd multiples of 1.23 ms) to evaluate the possible impact of lipid signals. The MR T2 and T2* maps were analyzed with ImageJ (http://rib.info.nih.gov). Regions of interest (ROIs) were placed within the bilateral anterior tibial muscles, soleus muscles, medial and lateral gastrocnemius muscles and regional fat tissues by hand tracing the shapes of the muscles in anatomic images, then copied and pasted onto the corresponding T2 and T2* maps. The T2 and T2* values for the healthy controls and DM subjects are presented as means ± standard errors of the mean (SEM).

Dixon's two point water-fat separation method, which provides information regarding total fat, including both intra- and extra-myocellular lipids, was applied to evaluate fat accumulation in the calf muscle region [20]. Images of water-fat in-phase and water-fat opposed-phase were acquired at TE values of 2.46 ms and 6.15 ms, respectively, and using the same slice locations and thickness used for the T2 and T2* measurements from nine T2DM subjects, ten young and healthy subjects, and seven healthy age-matched controls.

Fat and water maps were generated from the in-phase and out-of-phase images according to the following equations: fat = (in-phase−opposed-phase)/2 and water = (in-phase+opposed-phase)/2. The fat spatial ratio (FSR) = fat/(water+fat) was calculated and the value of FSR in the soleus was extracted in a similar manner to that used in the T2* extraction from the T2DM and healthy comparison subjects.

Group differences in demographic variables involving continuous and categorical data were calculated using independent t-tests and Fisher's exact test for a 2xk table, respectively. For comparison of T2* values in soleus, anterior tibialis, and gastrocnemius muscles, analysis of covariance (ANCOVA) or a multiple linear-regression method was used, controlling for age, BMI, and FSR. As there was a high co-linear correlation between HbA1c and group variables (namely HC vs DM)(r = 0.83, p<0.0001), we did not include HbA1c in the regression modeling. The normality assumption was verified for each variable of interest using the Shapiro-Wilk test. Statistical significance was defined at an alpha level of p<0.05, two tailed.

## Results

**Figure 1:**
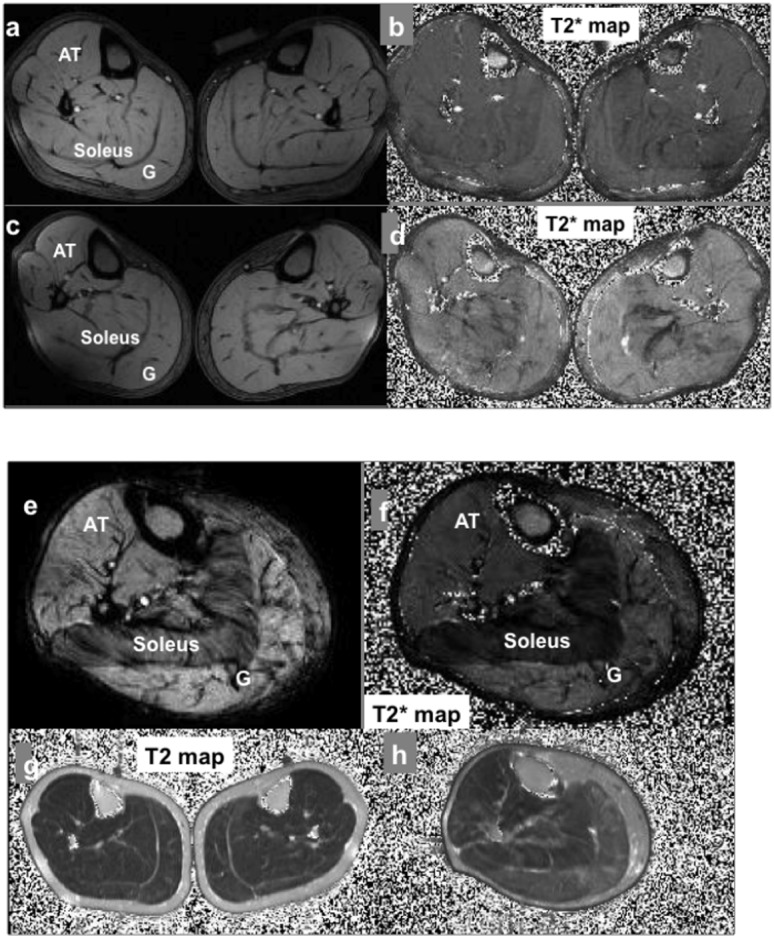
a–f T2* weighted images and T2* maps of calves of a young healthy control (a,b), a 64 year old T2DM subject (c,d), and a 68 year old T2DM subject (e,f) acquired at 3T. g–h representative T2 maps of healthy (g) and T2DM (h) calves. Due to relatively long T2s of the subcutaneous lipid and bone marrow (∼115 ms), the contrast among the muscles was less dramatic than that of T2*.

The images and corresponding T2 and T2* maps of the calf cross sections include anterior tibialis, soleus, gastrocnemius, and other muscles (Figures 1 a–h). T2DM soleus muscles displayed a noticeable hypointensity in comparison with those of the controls in the images and corresponding T2* maps (Figures 1 a,c,e vs b,d,f). No hypointense lesions in T1-weighted images or diffusive hyperintense lesions in T2-weighted images were found in the T2DM soleus muscle or other regions of the calf cross-sections [21]. The T2* fits had a slightly better linearity (R2≅0.998) compared to that of T2s (R2≅0.983). No significant difference in T2* values resulted from the fits of the echoes at in-phase and opposed-phase TEs.

**Figure 2:**
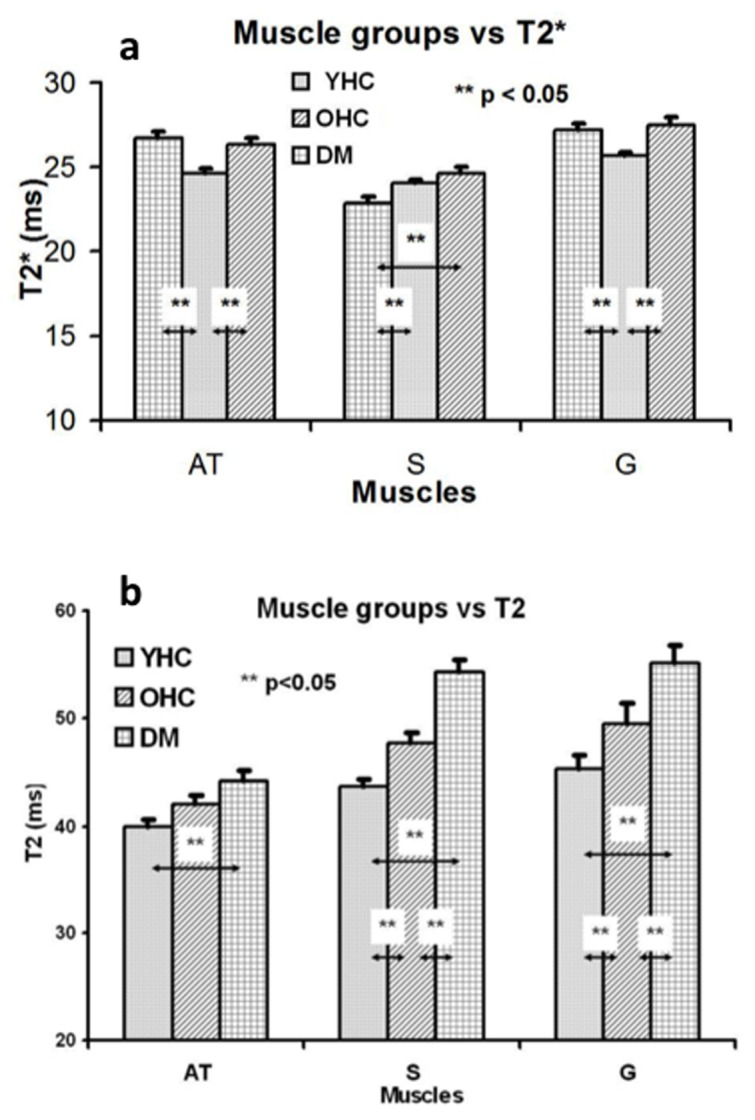
a. T2* distribution among anterior tibialis, soleus, and gastrocnemius of young healthy controls (YHC), older age-matched healthy controls (OHC), and T2DM subjects (DM). The error bars represent the standard errors of the means. The difference between the control and the T2DM soleus is significant. The difference between the AT and S is not significant in healthy controls, but it is in T2DM subjects. b. T2 distribution among anterior tibialis, soleus, and gastrocnemius of young healthy controls (YHC), older age-matched healthy controls (OHC), and T2DM (DM). The error bars represent the standard errors of the means. The difference between the control and the T2DM soleus is significant. Compared to T2*, the most dramatic difference is the much longer T2 of the diabetic soleus than observed in either the young or older healthy controls.

In healthy subjects, gastrocnemius muscles had a relatively high T2* value (25.3±0.6 ms) compared to soleus (23.9±0.5 ms) and anterior tibialis muscles (23.9±0.3 ms) (Figure 2a). The T2* values increase significantly in anterior tibialis and gastrocnemius as well as with age within the healthy control cohort (p = 0.01). However, the increase in T2* value did not reach statistical significance in the soleus of the healthy control subjects. In the calves of diabetic patients, both the anterior tibialis (AT) and gastrocnemius (G) had significantly higher T2* values (AT: 27.7±0.4 ms, G: 27.6±0.7 ms) than the soleus (S) (22.9±0.5 ms) (Figure 2a). The anterior tibialis T2* value was approximately 20% higher than that of the soleus. In across-group comparisons of healthy controls versus T2DM subjects, the soleus T2* values of T2DM subjects were significantly lower than both the young healthy controls (22.9±0.5 vs 23.9±0.3, p = 0.03) and the age-matched controls (22.9±0.5 vs 26.7±0.4, p<0.01). Note that, as expected, T2* values of subcutaneous fat and bone marrow were in the range of 30–40 ms, much higher than those of soleus in the T2DM cohort.

As a control measure in the skeletal muscles, T2 values of the calf cross-sections were also calculated. T2 maps of the calf cross-sections for the healthy controls and T2DM subjects showed a T2 value distribution pattern, T2 (G)≈T2 (soleus)>T2 (AT) (Figures 1 g&h Figure 2b). In the young, healthy subjects, T2 values for AT were 39.9±0.7 ms, for soleus 43.6±0.7 ms, and for gastrocnemius 45.2±0.8 ms. In the older healthy control group, the T2 of soleus, anterior tibialis and gastrocnemius was increased to 47.8±0.8 ms, 42.1±0.7 ms and 49.4±2.0 ms, respectively. T2 values of T2DM calves were significantly longer than those of the healthy controls (soleus: 54.3±5.7 ms; anterior tibialis: 44.2±4.0 ms, and gastrocnemius: 55.1±5.8 ms), consistent with a higher lipid content and a higher degree of fluid accumulation as measured independently with isotope dilution method in a separate study [22].

**Figure 3:**
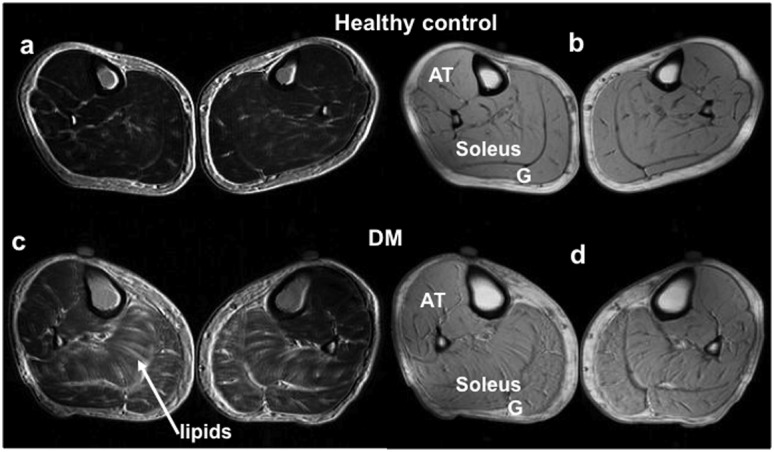
Representative maps of fat (out-of-phase) (a,c) and water (in-phase) (b,d) of healthy controls (a,b) and T2DM subjects (c,d). A dramatic increase of lipid can be seen in the diabetic soleus and gastrocnemius.

**Figure 4:**
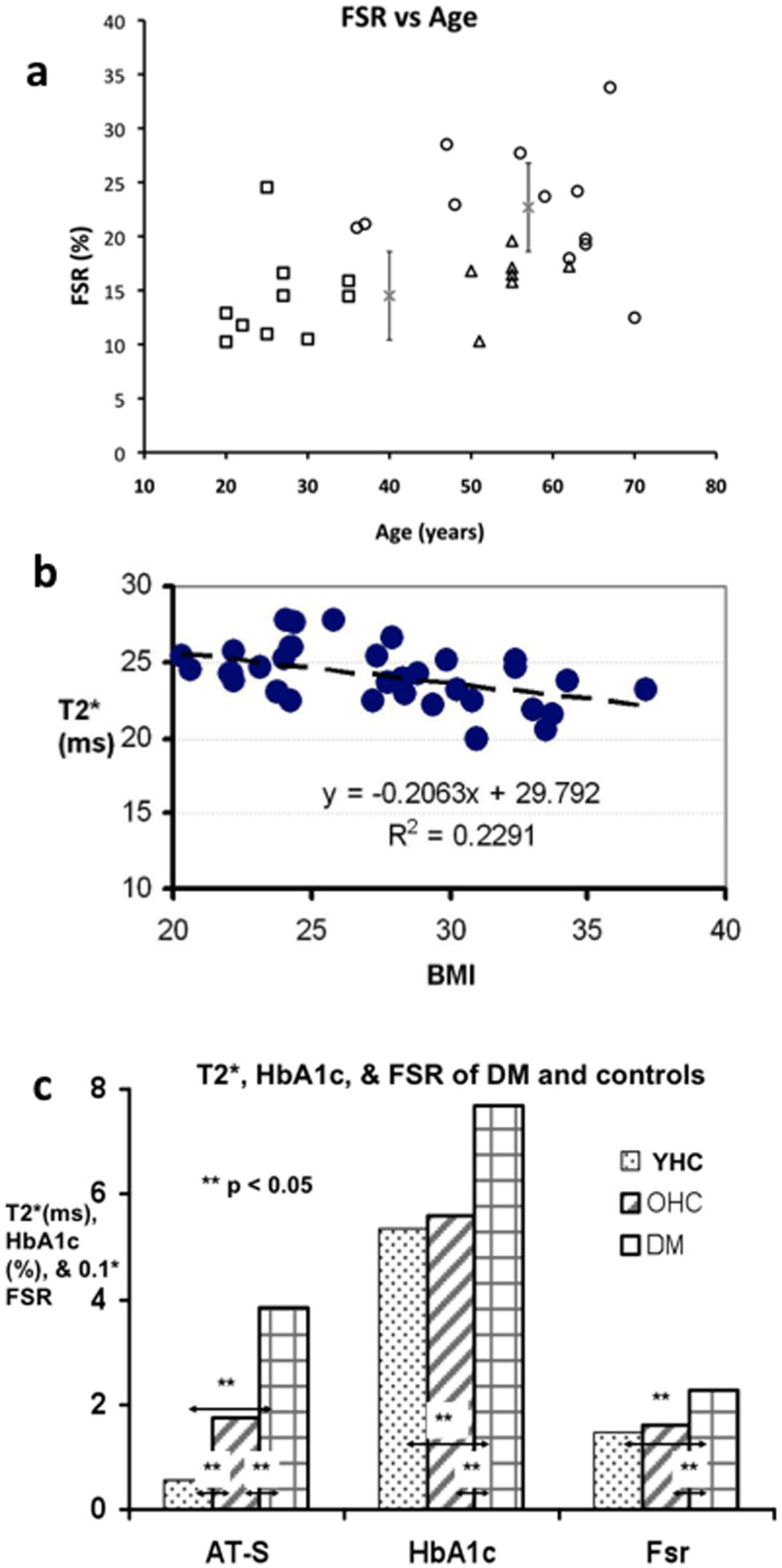
a Plot of FSR versus age for young (squares) and old (triangles) healthy controls as well as T2DM subjects (circles); x's represent the group averages of the DM and the healthy controls (both young and old) and the bars represent standard deviations. b Plot of soleus T2* versus BMI for healthy and T2DM subjects. c Bar plot of soleus T2* change versus anterior tibilias, HbA1c, and lipid index (FRS) for the young and older healthy controls (YHC and OHC) and T2DM (DM) subjects scanned in this study.

Lipid maps demonstrated that T2DM subjects had a dramatic increase in lipid deposition in the soleus and gastrocnemius muscles compared to the young and age-matched healthy control groups (Figures 3 a & c). Values of fat spatial ratio (FSR) were significantly higher in T2DM subjects than in the healthy controls, even when age effects were taken into account (Figure 4a). It is worth noting that subjects with a high FSR may have a normal soleus T2* value in the T2DM (e.g. FSR 33.8%, T2* 23.9 ms) and healthy control (e.g. FSR 24.5%, T2* 24.3 ms) groups. The aged-matched controls have a 2%-higher average FSR but a 2.4 ms-longer T2* compared to the young, healthy controls (p<0.02) (Figures 2a, 4a).

A weak relationship was found between FSR and age (Figure 4a) in young and age- matched healthy controls (Pearson coefficient 0.32) and between BMI and the soleus T2* values (Pearson coefficient −0.41) among all subjects (Figure 4b, r^2^ = 0.23 Slope −0.21). There was almost no FSR-age relationship among the T2DM subjects evaluated in this study (Pearson coefficient −0.11). Analysis of multiple linear regression indicates that a significant correlation existed between the soleus T2* values and the subject groups (healthy versus T2DM) (p<0.001) and between the T2* value and age (p = 0.03), but no significant correlation between the T2* values and BMI or FSR (p = 0.62 for BMI and 0.77 for FSR) (see table 2). This finding suggests that T2DM patients had significantly lower T2* values compared to healthy subjects, controlling for age. The regression coefficient −3.458 suggests a 13.1% T2* decrease compared to full T2* intensity in a diabetic subject after adjusting for age effects. The positive correlation coefficient between T2* and age (+0.058) indicates a mild increase in soleus T2* value with age, which is consistent with an increase of fluid accumulation (see Figure 2b).

**Table 2:** Summary of linear regression analysis between T2* and variables age, BMI, FSR, and group (HC = 0, DM = 1), where constant is the regression constant.

T2*	Coef.	std. err.	t	p>| t |
age	0.0577	0.0178	3.25	0.003
BMI	0.0552	0.0925	0.6	0.556
FSR	−0.0434	0.0585	−0.74	0.466
group	−3.4581	0.8259	−4.19	0.000
constant	22.3363	2.1785	10.29	0.000

Although their calf circumference was insignificantly larger than those of the controls (Table 1), the T2DM group had a significantly higher averaged HbA1c (7.7±0.3 mg/mL) compared to the young healthy controls (5.4±0.1 mg/mL) and the age-matched healthy controls (5.6±0.2 mg/mL) (Figure 4c). The correlation coefficient between T2* and subject groups (healthy control versus T2DM status) indicates a decrease in soleus T2* value as one progresses from healthy to the diabetic state (Table 2). The T2* values were also significantly negatively correlated with the HbA1c values (Pearson coefficient 0.55).

## Discussion

The major finding of this study is that at resting state, the soleus T2* value was significantly correlated with age and associated with the presence of diabetes (or elevated HbA1c) (see Table 2). The diabetic soleus T2* was abnormally short compared to the age-matched controls and the surrounding anterior tibialis and gastrocnemius (figures 2 and 4c). Underlying physiologic factors contribute to the T2 and T2* values of anterior tibialis, gastrocnemius, and soleus muscles of the subjects in this study. The T2 values were prolonged in anterior tibialis, soleus, and gastrocnemius of the T2DM and the age matched healthy controls reflecting increased fluid levels, and increased lipid levels mostly seen in T2DM, because of the disease and age-related propensity to accumulate fluid and lipid in the lower legs compared to the young, healthy individuals (Figure 2b). This explanation is consistent with the results measured independently by the isotope dilution method in a separate study [22] and is supported by the relatively long T2 values of subcutaneous fat. The longer T2* values in the anterior tibialis and gastrocnemius of T2DM subjects, as well as in the soleus muscles of older healthy subjects, were consistent with the prolonged T2 values due to accumulation of fluid and lipid.

The shorter T2* values in the soleus muscles of T2DM subjects (figures 1 and 2), however, may result from underlying pathophysiology despite the longer regional T2 value. It is known that gastrocnemius and anterior tibialis muscles are composed of predominantly fast-twitch fibers, while soleus consists largely of slow-twitch fibers [23]. Soleus is rich in mitochondria and capillary vessels and consumes more oxygen due to the characteristic energetic metabolism of slow-twitch fibers. Previous studies of animal muscles indicate that the diabetic state is associated with a reduction in capillaries capable of supporting red blood cell (RBC) perfusion [24], [25]. These muscle capillary defects have been confirmed in diabetic patients by thallium-201 scanning [26]. Interestingly, Cosson et al found the calf muscle perfusion defects were also significantly correlated with elevated HbA1c levels, similar to our findings (Table 2). Therefore, the reduced perfusion in the microvascular bed of the soleus muscle could lead to an increase in regional deoxyhemoglobin levels and unusually low regional T2* compared to gastrocnemius and anterior tibialis. This is consistent with the present findings.

Abnormally low soleus T2* values in diabetic calf muscle have been observed in a previous study. Ledermann et al applied a cuff compression paradigm for reactive hyperemia and BOLD MRI with four-echo EPI to patients with peripheral arterial occlusive disease (PAOD) and found soleus had the larger T2* changes (ΔT2*) and longer time-to-peak compared to anterior tibialis and gastrocnemius after cuff deflation [27]. Although they did not report resting state T2* values for the soleus, the large ΔT2* implied a lower soleus T2* among the calf muscles at baseline, consistent with the findings of the current study.

In addition to the lower soleus T2*, lipid significantly accumulated, and was noticeably visible in the MR images as a thickening subcutaneous fat layer, with infiltration into the gastrocnemius and soleus of the T2DM subjects (Figures 1 and 3). This characteristic of T2DM skeletal muscle likely resulted from insulin resistance, impaired glycogen synthesis, and impairments in mitochondria. Nonetheless, the non-significant correlation between soleus T2* and regional lipid levels, as illustrated in Table 2, suggests that the T2DM state (or elevated HbA1c) is the dominant contributor to the abnormally low soleus T2* value.

Compared to muscle exercise and kinetic paradigms, resting state studies use a lower temporal resolution and a higher spatial resolution to measure the T2* values in calf muscles. This imaging scheme is simple, short in duration (less than 7 minutes for acquisitions of both T2 and T2* images), and more acceptable to patients with clinical disabilities and may thus have broader utility in applications that require high spatial resolution. The resting state approach may provide complementary information regarding regional tissue oxygen consumption, to perfusion studies in nuclear medicine [26] or to exercise and kinetic paradigms [27], [28]. Moreover, these MRI techniques may provide information that allows clinicians to avoid a potentially harmful biopsy and serve as a valuable diagnostic alternative for diabetic patients with vascular complications, since many of these patients may have coexisting diabetic nephropathy, to reduce the risk associated with the administration of gadolinium-based contrast agents. Clinical use of these techniques may provide valuable information for investigating and clinically evaluating the systemic microvascular and macrovascular changes that lead to the more well-recognized diabetic complications such as retinopathy, neuropathy, or cardiovascular disease.

In summary, we have measured T2 and T2* values of calf muscles at rest and found that soleus T2* is significantly correlated with age, the presence of T2DM or elevated HbA1c. The T2* of T2DM soleus muscle was significantly lower than that of age matched healthy controls and that of young and healthy controls as well as the T2* values of T2DM anterior tibialis and gastrocnemius. Our data indicate that the soleus T2* measures provide valuable information reflecting underlying physiology and are consistent with previous findings of other studies. Among factors that may contribute to T2* values, the lowered T2* in the T2DM soleus muscle is most consistent with a combination of high oxygen consumption and poor regional microvascular circulation which suggests that the T2DM soleus is likely under tissue oxygenation stress.
